# Detection of paralytic shellfish toxins and tetrodotoxins in shellfish using a single-cell biosensor based on patch clamp technology

**DOI:** 10.1007/s00204-026-04437-5

**Published:** 2026-05-13

**Authors:** Jaume Reverté, Andrew D. Turner, Monika Dhanji-Rapkova, Mirjam D. Klijnstra, Arjen Gerssen, Sandra Lage, Àngels Tudó, Jorge Diogène, Francesc X. Sureda, Mònica Campàs

**Affiliations:** 1 IRTA, Marine and Continental Waters (AMiC), La Ràpita, 34540 Catalonia Spain; 2 Facultat de Medicina i Ciències de la Salut, Departament de Ciències Mèdiques Bàsiques, Universitat Rovira i Virgili (URV), Reus, 43201 Spain; 3 Centre for Environment Fisheries and Aquaculture Science (CEFAS), Barrack Road, Weymouth, DT4 8UB UK; 4 Wageningen Food Safety Research, Wageningen University & Research, Akkermaalsbos 2, Wageningen, 6708 WB The Netherlands; 5 Centro de Ciências do Mar do Algarve (CCMAR/CIMAR LA), Universidade do Algarve, Campus de Gambelas, Faro, 8005-139 Portugal

**Keywords:** Automated patch clamp, Electrophysiology, Toxicology, Neurotoxin, Food safety

## Abstract

Paralytic shellfish toxins (PSTs) are a well-known group of potent neurotoxins that may accumulate in shellfish, posing a significant risk to food safety and public health. To protect consumers, shellfish production areas are subject to regulatory monitoring programs targeting PST contamination. However, tetrodotoxins (TTXs), another group of potent neurotoxins that may also accumulate in shellfish and co-occur with PSTs, are not currently included in regulations and routine monitoring schemes in most EU countries. This is of particular concern because PSTs and TTXs share the same biological target and mechanism of action and therefore pose a comparable neurotoxic risk. In this work, we present an automated patch clamp (APC) single-cell biosensing device as a toxicological bioanalytical solution addressing the need for tools capable of simultaneously detecting these two hazardous toxin groups. The biosensor was able to detect not only saxitoxin (STX) and TTX, but also their toxic analogues. The method achieved a limit of detection of 37 µg STX equivalents (equiv.) kg⁻¹, well below the current regulatory limit of 800 µg STX equiv. kg⁻¹. After the analysis of more than 70 samples exhibiting diverse toxin profiles, the results and correlations with reference methods demonstrate that APC single-cell biosensing provides a robust and integrative tool for the simultaneous monitoring of PSTs and TTXs.

## Introduction

The global demand for seafood is expected to increase in the coming decades, with shellfish projected to play a critical role in meeting this growing need (Food and Agriculture Organisation (FAO) of the United Nations 2024). This importance stems not only from their value as a healthy and nutritious source of animal protein (Venugopal and Gopakumar 2017), but also from their high production efficiency and relatively low environmental footprint compared with other food production systems (Esposito et al. 2026). However, the filter-feeding nature of shellfish renders them particularly susceptible to marine toxins, which can accumulate in their tissues, posing significant risks to food safety and public health (Reverté et al. 2023). One of the seafood-borne illnesses is paralytic shellfish poisoning (PSP), caused by the consumption of shellfish contaminated with saxitoxin (STX) and its analogues, collectively referred to as paralytic shellfish toxins (PSTs) (Deng et al. 2025).

In marine ecosystems, the production of PSTs is generally associated with harmful algal blooms (HABs) of toxic dinoflagellates of the genera *Alexandrium*, *Gymnodinium*, and *Pyrodinium* (Xu et al. 2026). The toxicity of these compounds arises from their specific blockage of voltage-gated sodium channels (VGSCs), which are key cell membrane proteins involved in the initiation and propagation of action potentials (Mackieh et al. 2021). Clinical manifestations of PSP commonly include paranesthesia of the lips, face, arms, and legs, as well as ataxia, muscle weakness, nausea, and vomiting. More severe symptoms, such as dizziness or vertigo, dysarthria, paralysis, and respiratory distress, have also been reported across diverse geographical regions (Gribble et al. 2025). Management of PSP is largely supportive and focused on symptom relief; however, without prompt intervention, the condition can result fatal (Temple and Hughes 2021). To protect public health, shellfish production areas are subject to rigorous regulatory monitoring programs that enforce harvesting closures when toxin concentrations exceed established safety thresholds; in the case of PSTs, 800 µg STX equivalents (equiv.) kg⁻¹ (Regulation (EC) No 853/2004). Although these monitoring strategies have been effective in substantially reducing the global incidence of PSP (Etheridge 2010), this status could be challenged by the emergence of tetrodotoxins (TTXs) in shellfish.

Historically, TTXs have been considered exclusive to pufferfish (Miyazawa and Noguchi 2021). However, due to their bacterial origin (Magarlamov et al. 2017), they can also be found in other marine organisms (Bane et al. 2014), including shellfish (Biessy et al. 2019; Turner et al. 2015; Vlamis et al. 2015). The presence of TTXs in shellfish is particularly concerning because, in contrast with other toxins that also accumulate in shellfish, TTX has the peculiarity to share the same biological target and toxicity mechanism as PSTs (Mackieh et al. 2021). In fact, the symptoms caused by TTX are very similar to those produced by PSTs (Katikou et al. 2022). Despite the toxic similarities and the comparable risk that both toxins pose to human health (Finch et al. 2018), TTX in shellfish is not currently subject to the same regulatory monitoring requirements as PSTs in many parts of the world. This discrepancy is problematic, as the co-occurrence of PSTs and TTXs in shellfish has been increasingly reported worldwide (Hwang et al. 1995; Jen et al. 2007, 2014; Dell’Aversano et al. 2019; Numano et al. 2019; Turner et al. 2020; Shingai et al. 2024). Under these scenarios of toxin co-occurrence, single-toxin evaluations may underestimate the overall neurotoxic risk associated with the consumption of contaminated shellfish. Therefore, the application of integrated analysis strategies for the simultaneous monitoring of PSTs and TTXs should be considered for a proper food safety assessment.

The development of immunosensing tools for the simultaneous detection of PSTs and TTXs in seafood could be of particular interest. Biosensors have demonstrated strong performance and offer several advantages over conventional instrumental analytical methods used for toxin monitoring, including the absence of requirements for sophisticated equipment, centralised laboratories, and specialised personnel, as well as straightforward data interpretation compatible with rapid risk-management decision-making. In this regard, anti-TTX antibodies have shown the ability to detect TTX analogues in proportion to their toxicity (Reverté et al. 2026), supporting their suitability for comprehensive toxin detection. In contrast, the limited cross-reactivity of anti-STX antibodies towards certain toxic STX analogues (Aballay-González et al. 2025) represents a significant limitation, as these hazardous compounds may escape detection. In this context, toxicological biosensing approaches for the combined detection of STX, TTX, and their analogues represent a promising alternative, as they provide an integrated assessment of overall sample toxicity, capturing the combined effects of all toxins present.

In this work, we present an automated patch clamp (APC) single-cell biosensing device as a bioanalytical tool for the simultaneous detection of PSTs and TTXs in shellfish. The biosensing approach is based on assessing the toxic effects of these compounds on Neuro-2a cells by measuring the inhibition of VGSC currents resulting from toxin-channel interactions. To validate the system, we first assessed its ability to detect STX, TTX, and their analogues. Then, we evaluated the impact of shellfish matrix components on toxin detection and implemented strategies to minimise their interferences. Finally, the biosensor was applied to over 70 contaminated shellfish samples, including samples with confirmed co-occurrence of PSTs and TTXs, and the results were benchmarked against reference instrumental analysis methods. Our main objective was to demonstrate the potential of APC single-cell biosensing as an integrative tool for monitoring toxins that share a common mechanism of action.

## Materials and methods

### Toxin standards

Certified reference standards of STX and its analogues, including decarbamoyl saxitoxin (dcSTX), neosaxitoxin (NEO), decarbamoyl neosaxitoxin (dcNEO), N-sulfocarbamoyl gonyautoxin 1 and 2 (C1&2), N-sulfocarbamoyl gonyautoxin 3 and 4 (C3&4), gonyautoxin 1 and 4 (GTX1&4), gonyautoxin 2 and 3 (GTX2&3), decarbamoyl gonyautoxin 2 and 3 (dcGTX2&3), gonyautoxin 5 (GTX5), and gonyautoxin 6 (GTX6), were obtained from CIFGA (Lugo, Spain). TTX was purchased from Tocris Bioscience (Bristol, United Kingdom) and the standard solution was prepared at 1 mg mL⁻¹ with acetic acid at 1% (*v*/*v*).

## Shellfish samples and toxin extraction

A collection of shellfish samples, including bivalves (i.e., oysters, mussels, clams, razor clams, geoducks and cockles) and gastropods (i.e., marine snails), collected between 2016 and 2024 from different European countries (United Kingdom, Netherlands and Portugal, among others) and Mexico, were used for the validation of the APC single-cell biosensor. All samples were captured within the framework of nationally or EU-supported monitoring programs or research projects, in compliance with the relevant ethical guidelines established by the competent authorities. Upon arrival at the laboratory the samples were processed and stored at – 20 °C until extraction. For bivalves, the shells were opened and the whole soft tissues recollected. Regarding gastropods, they were dissected, and their tissues were separated into flesh (i.e., cerebral ganglia, foot muscle, mantle, mouth/proboscis and salivary glands) and visceral (i.e., anus/rectum, digestive gland, gill, heart, intestine, kidney, and stomach) parts.

Since both PSTs and TTXs are hydrophilic toxins, the same extraction protocol was applied to all shellfish samples (Campàs et al. 2020). Briefly, 1 g of shellfish tissue was weighed into tubes and homogenised using an Ultra-Turrax blender at maximum speed. To each tube, 1 mL of 1% (*v*/*v*) acetic acid was added, followed by vortexing for 5 min at 2500 rpm. The tubes were then placed in a boiling water bath for 10 min with occasional stirring. After cooling to room temperature, samples were centrifuged at 2500 rpm for 5 min at 4 °C, and the resulting supernatants were collected. In the case of the gastropods, an additional clean-up step using solid phase extraction (SPE) was applied (Pais et al. 2025). Following filtration, the final extracts, corresponding to 1000 mg tissue equiv. mL⁻¹, were stored at − 20 °C until analysis.

## Automated patch clamp (APC) single-cell biosensor analysis

The analysis of shellfish with the APC single-cell biosensing device was based on a previously described protocol for TTX detection in pufferfish using the Patchliner platform (Nanion Technologies GmbH, Munich, Germany) (Campàs et al. 2024). Briefly, a suspension of Neuro-2a cells, prepared at 100,000 cells mL⁻¹ in a 1:1 mixture of Roswell Park Memorial Institute (RPMI-1640) medium and external buffer, was injected into medium resistance borosilicate chips previously filled up with internal (50 mM CsCl, 10 nM NaCl, 60 mM CsF, 20 nM EGTA, 10 mM HEPES/CsOH, pH 7.2) and external (140 mM NaCl, 4 mM KCl, 1 mM MgCl_2_, 2mM CaCl_2_, 5 mM D-glucose monohydrate, 10 mM HEPES/NaOH, pH 7.4) solutions. Each chip well consists of two fluidic chambers (internal and external) interconnected by a small aperture. A single cell was captured in this aperture by applying − 50 mbar of pressure. After cell immobilisation, an enhancer solution (10 mM CaCl₂, 10 mM MgCl₂, pH 7.4, osmolarity 302 mOsmol) was added, and the membrane potential was set to − 100 mV. Negative pressure was then gradually increased from − 50 to − 300 mbar until the whole-cell configuration was achieved. Successful patching was confirmed by the formation of a stable gigaohm seal, with resistances exceeding 1 GΩ. The changes in sodium currents by the effect of the toxins were measured at − 100 mV during 40 s after injection using an EPC Quatro USB amplifier unit with a 4-probe configuration from HEKA Elektronik (Stuttgart, Germany), which were controlled and digitalised in real time with the Patchmaster software (Nanion Technologies GmbH, Munich, Germany).

For biosensor analysis, toxin standards and samples were tested following the same protocol. In all cases, twelve successive 1:2 serial dilutions were prepared in external solution and injected into the APC biosensor. The maximum concentrations tested were 150 nM for STX, 150 nM for TTX, and 75 nM STX + 75 nM TTX for the mixture experiment. For STX analogues, the maximum concentrations tested were 3038 nM for dcSTX, 101 nM for NEO, 28,970 nM for dcNEO, 10,517 nM for C1&2, 10,175 nM for C3&4, 243 nM for GTX1&4, 395 nM for GTX2&3, 7096 nM for dcGTX2&3, 26,361 nM for GTX5, and 12,647 nM for GTX6. For the study of the non-specific blockage of the sodium currents by the shellfish matrix, the highest extract concentration tested was 1000 mg tissue equiv. mL⁻¹. For the study of the inhibition of toxin-target interaction by the shellfish matrix, twelve 1:2 serial dilutions of 150 mM STX were prepared in external buffer containing 10 mg tissue equiv. mL⁻¹ of blank mussel, oyster, and marine snail (flesh and viscera) extract. For the analysis of samples, the highest shellfish concentration tested for each one of the extracts was 10 mg tissue equiv. mL⁻¹; when necessary, sample extracts were further diluted to ensure quantification.

## Results

### Detection of STX and TTX with the APC biosensor

**Fig. 1:**
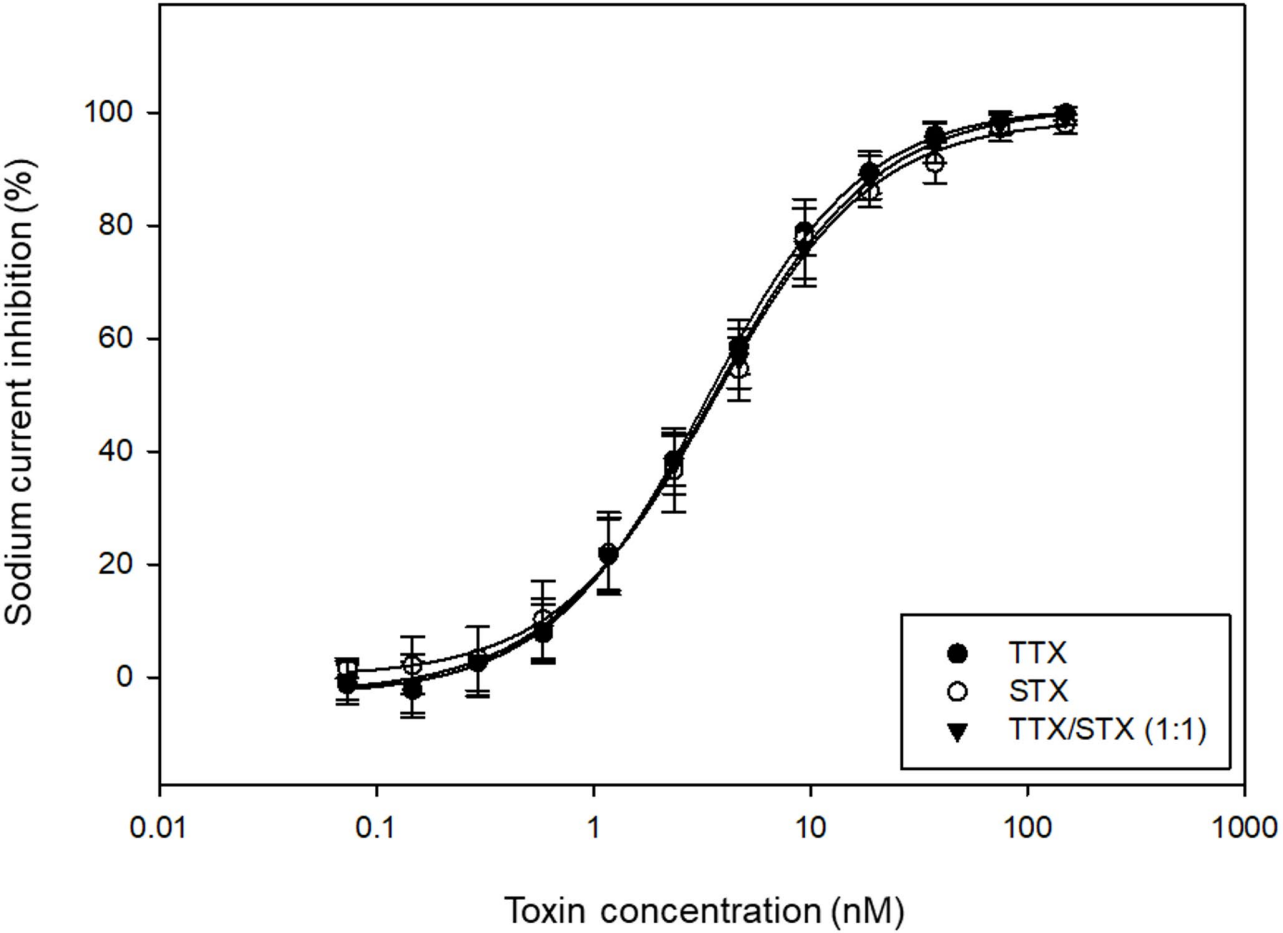
Inhibition of sodium currents in Neuro-2a cells by TTX, STX, and a 1:1 molar ratio TTX/STX mixture. Biosensor data were normalised with respect to the maximum current of each individual cell and fitted to a sigmoidal logistic four-parameter model. Each point represents the average ± standard deviation (*n* = 4)

The individual and combined toxicity of STX and TTX were evaluated at electrophysiological level by monitoring the inhibition of sodium currents in Neuro-2a cells using the APC single-cell biosensing device. Whether tested individually or in a 1:1 molar ratio combination, both toxins produced comparable concentration-dependent inhibition curves (Fig. 1), exhibiting identical half-maximal inhibitory concentrations (IC_50_) and highly similar working ranges (IC_20_ – IC_80_). These results indicate that, in Neuro-2a cells, the toxicity of STX and TTX is equivalent and additive. Based on this observation, the regulatory limit of 800 µg STX equiv. kg⁻¹ (Regulation (EC) No 853/2004) was adopted in this work as the threshold for determining whether samples were safe for human consumption.

### Toxicity equivalency factors for STX and TTX analogues

**Fig. 2:**
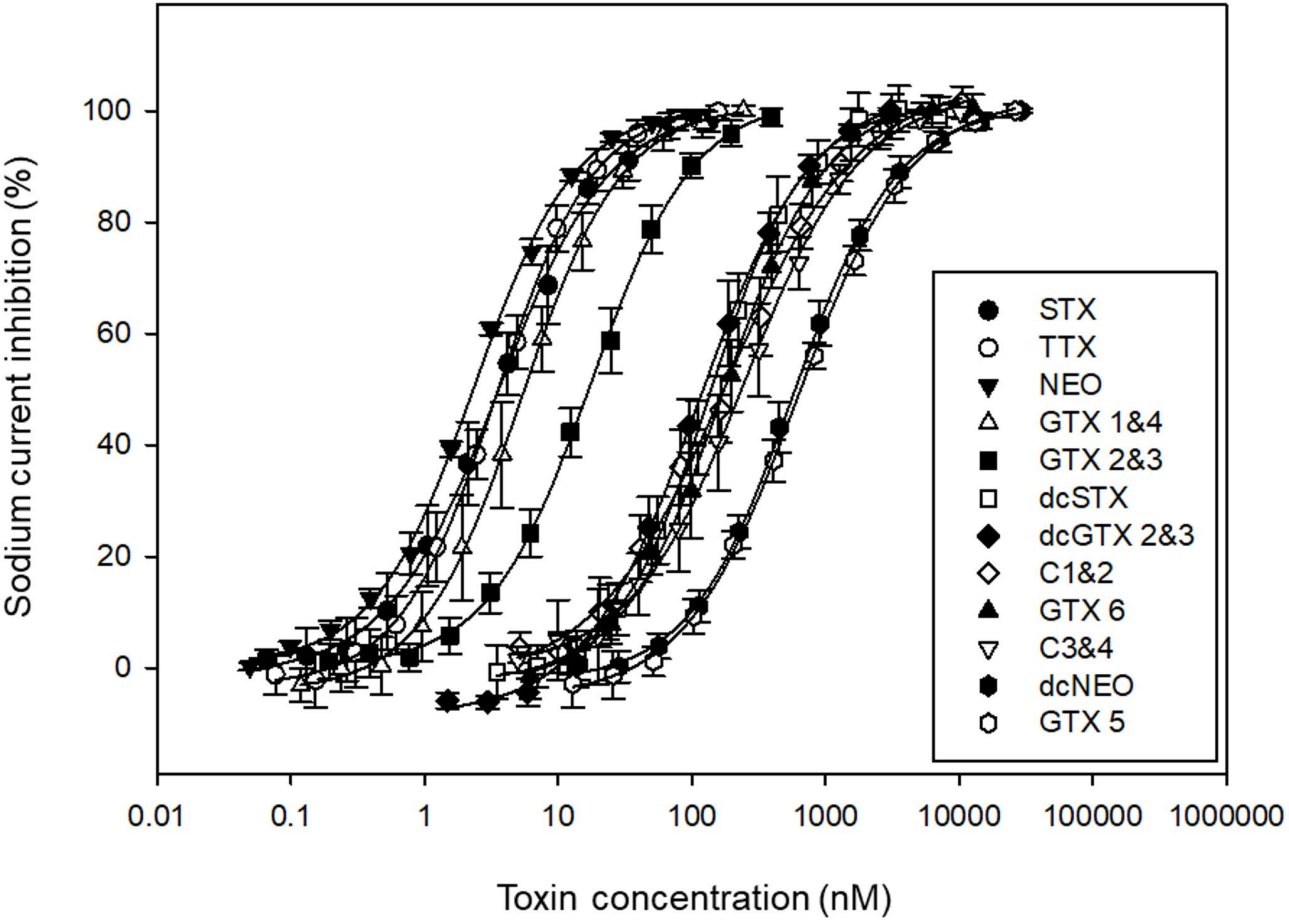
Inhibition of sodium currents in Neuro-2a cells by PSTs and TTX. Biosensor data were normalised with respect to the maximum current of each individual cell and fitted to a sigmoidal logistic four-parameter model. Each point represents the average ± standard deviation (*n* = 4)

The individual toxicities of dcSTX, NEO, C1&2, C3&4, GTX1&4, GTX2&3, dcGTX2&3, GTX5, and GTX6 at electrophysiological level were evaluated by monitoring the inhibition of sodium currents in Neuro-2a cells using the APC single-cell biosensing device. Then, the toxicity equivalency factors (TEFs) were calculated as the ratio between the IC_50_ value of STX to that of each STX analogue. As shown in Fig. 2, all STX analogues induced a concentration-dependent inhibition of sodium currents in Neuro-2a cells. However, their toxic potencies were not equivalent to that of STX. Among the tested STX analogues, NEO was the most potent (TEF_APC_ = 1.546), followed by GTX1&4, GTX2&3, dcSTX, dcGTX2&3, C1&2, GTX6, dcNEO, and GTX5, all with TEF_APC_ < 1 (Table 1).

**Table 1:** Half-maximal inhibitory concentrations (IC_50_) derived from the Neuro-2a sodium current inhibition curves of PSTs and TTX along with the calculated toxicity equivalency factors (TEFs).

Toxin	IC_50_ (nM)	TEF_APC_	TEF_EFSA_	TEF_FAO/WHO_
**STX**	4	1	1	1
**TTX**	4	1	-	-
**NEO**	2	1.546	1.0	2.0
**GTX1&4**	6	0.639	1.0 (GTX1) 0.7 (GTX4)	1.0 (GTX1) 0.7 (GTX4)
**GTX2&3**	17	0.214	0.4 (GTX2) 0.6 (GTX3)	0.4 (GTX2) 0.6 (GTX3)
**dcSTX**	123	0.029	1.0	0.5
**dcGTX2&3**	140	0.026	0.2 (dcGTX2) 0.4 (dcGTX3)	0.2 (dcGTX2) 0.4 (dcGTX3)
**C1&2**	174	0.021	- (C1) 0.1 (C2)	0.01 (C1) 0.1 (C2)
**GTX6**	175	0.021	0.1	0.05
**C3&4**	236	0.015	- (C3) 0.1 (C4)	0.01 (C3) 0.1 (C4)
**dcNEO**	601	0.006	0.4	0.2
**GTX5**	653	0.006	0.1	0.1

The TEFs from EFSA and FAO/WHO are also presented for comparative purposes

The TEF_APC_ values for TTX analogues were previously determined in Neuro-2a cells using the same APC single-cell biosensor applied in this study (Reverté et al. 2024). Because TTX and STX have equivalent toxicity, using STX instead of TTX as the reference compound does not alter the previously established TEF_APC_ values. Therefore, the TEF_APC_ values remain as reported by Reverté et al. (2024): 0.238 for 11-norTTX-6(*S*)-ol, 0.107 for 11-deoxyTTX, 0.035 for 6,11-dideoxyTTX, 0.027 for 5,11-dideoxyTTX, and 0.001 for 5,6,11-trideoxyTTX. These results demonstrate that, in addition to STX and TTX, the biosensor can also detect their toxic analogues.

## Evaluation of shellfish matrix interferences on APC recordings

**Fig. 3:**
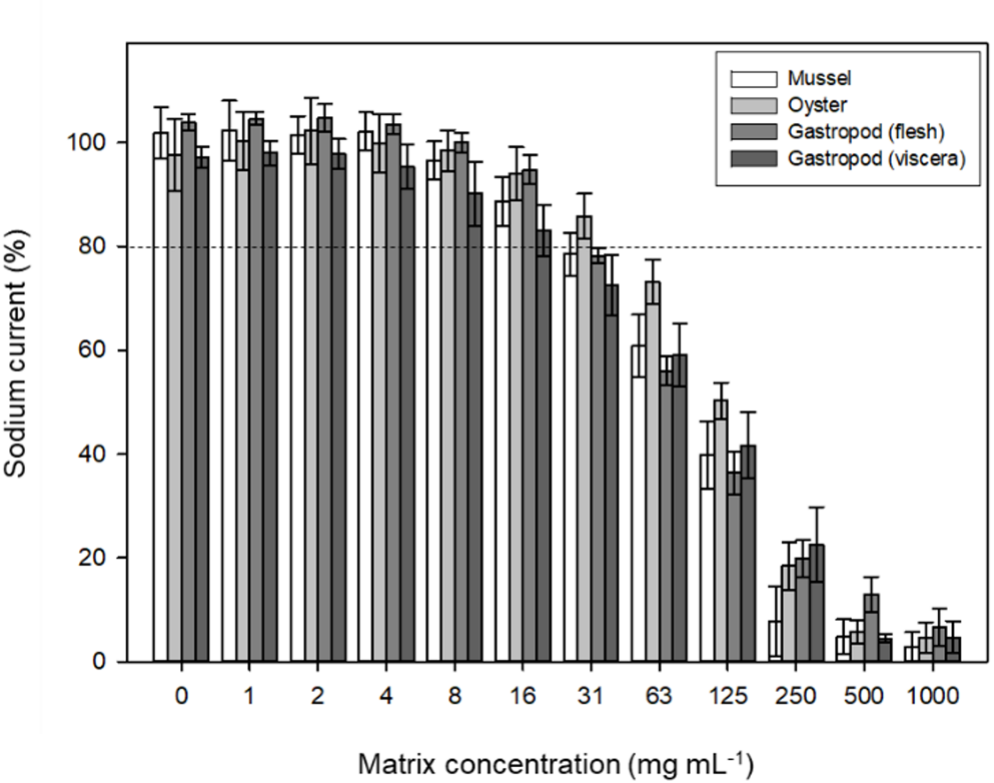
Non-specific inhibition of sodium currents in Neuro-2a cells by mussel, oyster, and marine snail (flesh and viscera) extracts. Biosensor data were normalised with respect to the maximum current of each individual cell. Each bar represents the average ± standard deviation (*n* = 4)

To evaluate the potential non-specific blockage of VGSCs by shellfish matrix components (i.e., sodium currents < 80%), Neuro-2a cells were perfused with varying concentrations of blank mussel and oyster extracts, confirmed to be free of PSTs and TTXs using instrumental analysis techniques. Marine snail extracts from both flesh and viscera tissues were also evaluated. While the flesh extract was confirmed to be toxin-free, no blank viscera was available; therefore, a viscera extract containing only trace levels of TTXs was used instead. As illustrated in Fig. 3, no matrix effects were observed at shellfish tissue concentrations up to 16 mg mL⁻¹ in the mussel sample (S45, sodium current of 89%) and up to 31 mg mL⁻¹ in the oyster sample (S32, sodium current of 86%). Regarding marine snails, no matrix effects were observed at 16 mg mL⁻¹ for both flesh (S66, sodium current of 95%) and viscera (S64, sodium current of 83%) samples. Based on these observations, we established that a conservative shellfish tissue concentration of 10 mg mL⁻¹ should not induce any non-specific blockage of the sodium currents in Neuro-2a cells. Accordingly, all shellfish samples should be diluted at least 200-fold before analysis to avoid false-positive results.

**Fig. 4:**
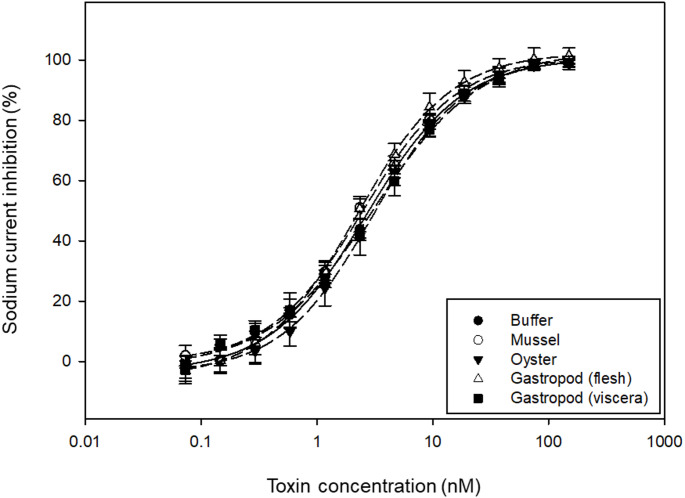
Inhibition of sodium currents in Neuro-2a cells by STX in presence of mussel, oyster, and marine snail (flesh and viscera) matrices at a shellfish tissue concentration of 10 mg mL⁻¹. Biosensor data were normalised with respect to the maximum current of each individual cell and fitted to a sigmoidal logistic four-parameter model. Each point represents the average ± standard deviation (*n* = 4)

To assess whether shellfish matrix components influence toxin–VGSC binding, the same mussel, oyster, and marine snail flesh and viscera extracts at 10 mg mL⁻¹ were spiked at different STX concentrations, and dose-response curves were constructed. As illustrated in Fig. 4, the deviation of the STX concentration-response curves obtained in presence of the mussel, oyster and marine snail matrices was minimal compared with the STX curve prepared in buffer, with toxin recoveries ranging between 84 and 106%. These results indicate that, under the specified conditions, shellfish matrix components do not significantly hinder the toxin-target interaction and therefore, by analysing the samples at a tissue equiv. concentration of 10 mg mL⁻¹ or lower, the biosensor avoids not only false-positive but also false-negative results.

Following the assessment of matrix effects, and under the established analytical conditions limiting the amount of shellfish matrix introduced into the system to 10 mg mL⁻¹ in order to minimise matrix-induced interferences, the biosensor achieved a limit of detection (LOD), defined as three times the standard deviation of the blank, of 11 µg STX equiv. kg⁻¹, and a limit of quantification (LOQ), defined as ten times the standard deviation of the blank, of 37 µg STX equiv. kg⁻¹. Based on these analytical parameters and considering that STX and TTX exhibit comparable toxicity in Neuro-2a cells, the APC single-cell biosensor showed to be sensitive enough to detect PSTs and TTXs at the proposed threshold of 800 µg STX equiv. kg⁻¹.

## Analysis of shellfish samples with the APC biosensor

A total of 73 shellfish samples collected from several European regions and Mexico were analysed using the APC single-cell biosensing device as part of an intralaboratory validation trial. The results are presented in Tables 2, 3, 4 and 5. The tables also include analytical data obtained from previous works, in which the shellfish samples were analysed with instrumental techniques (i.e., liquid chromatography coupled to mass spectrometry (LC-MS/MS) or liquid chromatography coupled to high resolution mass spectrometry (LC-HRMS) (Turner et al. 2020; Alkassar et al. 2024b; Pais et al. 2025). The corresponding TEFs were applied for comparison with the biosensor responses. Oysters were the most abundant shellfish type (*n* = 40, S01–S40; Table 2), followed by mussels (*n* = 14, S41–S54; Table 3) and marine snails (*n* = 14, S55–S68; Table 4). The remaining samples were less represented and included clams (*n* = 2, S69 and S71), cockles (*n* = 1, S70), geoducks (*n* = 1, S72), and razor clams (*n* = 1, S73) (Table 5). Among the different groups, the viscera from marine snails exhibited the highest toxicities, with toxin contents ranging from 1075 to 17,794 µg STX equiv. kg⁻¹. In contrast, the toxin contents in oysters (37–1003 µg STX equiv. kg⁻¹), mussels (350–4032 µg STX equiv. kg⁻¹), and other shellfish species (122–1365 µg STX equiv. kg⁻¹) were within a comparable range and substantially lower than those observed in marine snails.

**Table 2:** Concentration of PSTs/TTXs in oyster samples determined with the APC single-cell biosensor and LC-MS/MS.

Sample	Species	Biosensor	LC-MS/MS (with TEFs)		
			∑PSTs + TTXs	∑PSTs	∑TTXs
S01	*Crassostrea gigas*	1095	1003	885	119
S02	*Crassostrea gigas*	563	797	< LOQ	797
S03	*Crassostrea gigas*	381	299	299	< LOQ
S04	*Crassostrea gigas*	340	332	< LOQ	332
S05	*Crassostrea gigas*	243	181	< LOQ	181
S06	*Crassostrea gigas*	168	171	< LOQ	171
S07	*Crassostrea gigas*	134	132	< LOQ	132
S08	*Crassostrea gigas*	143	138	< LOQ	138
S09	*Crassostrea gigas*	135	123	< LOQ	123
S10	*Crassostrea gigas*	98	97	< LOQ	97
S11	*Crassostrea gigas*	92	106	< LOQ	106
S12	*Crassostrea gigas*	87	81	< LOQ	81
S13	*Crassostrea gigas*	80	97	< LOQ	97
S14	*Crassostrea gigas*	73	78	< LOQ	78
S15	*Crassostrea gigas*	59	50	< LOQ	50
S16	*Crassostrea gigas*	58	70	< LOQ	70
S17	*Crassostrea gigas*	55	50	< LOQ	50
S18	*Crassostrea gigas*	41	58	< LOQ	58
S19	*Crassostrea gigas*	38	44	< LOQ	44
S20	*Crassostrea gigas*	37	39	< LOQ	39
S21	*Crassostrea gigas*	< LOQ	31	< LOQ	31
S22	*Crassostrea gigas*	< LOQ	30	< LOQ	30
S23	*Crassostrea gigas*	< LOQ	30	< LOQ	30
S24	*Crassostrea gigas*	< LOQ	30	< LOQ	30
S25	*Crassostrea gigas*	< LOQ	30	< LOQ	30
S26	*Crassostrea gigas*	< LOQ	27	< LOQ	27
S27	*Crassostrea gigas*	< LOQ	26	< LOQ	26
S28	*Crassostrea gigas*	< LOQ	26	< LOQ	26
S29	*Crassostrea gigas*	< LOQ	14	< LOQ	14
S30	*Crassostrea gigas*	< LOQ	6	< LOQ	6
S31	*Crassostrea gigas*	< LOQ	3	< LOQ	3
S32	*Crassostrea gigas*	< LOQ	< LOQ	< LOQ	< LOQ
S33	*Crassostrea gigas*	< LOQ	< LOQ	< LOQ	< LOQ
S34	*Crassostrea gigas*	< LOQ	< LOQ	< LOQ	< LOQ
S35	*Crassostrea gigas*	< LOQ	< LOQ	< LOQ	< LOQ
S36	*Crassostrea gigas*	< LOQ	< LOQ	< LOQ	< LOQ
S37	*Crassostrea gigas*	< LOQ	< LOQ	< LOQ	< LOQ
S38	*Crassostrea gigas*	< LOQ	< LOQ	< LOQ	< LOQ
S39	*Crassostrea gigas*	< LOQ	< LOQ	< LOQ	< LOQ
S40	*Crassostrea gigas*	< LOQ	< LOQ	< LOQ	< LOQ

Concentrations are expressed as µg STX equiv. kg⁻¹

**Table 3:** Concentration of PSTs/TTXs in mussel samples determined with the APC single-cell biosensor and LC-MS/MS.

Sample	Species	Biosensor	LC-MS/MS (with TEFs)		
			∑PSTs + TTXs	∑PSTs	∑TTXs
S41	*Mytilus edulis*	4032	3904	3904	< LOQ
S42	*Mytilus edulis*	3335	3720	3700	21
S43	*Mytilus edulis*	2511	2705	2683	21
S44	*Mytilus edulis*	350	377	377	< LOQ
S45	*Mytilus edulis*	< LOQ	< LOQ	< LOQ	< LOQ
S46	*Mytilus edulis*	< LOQ	< LOQ	< LOQ	< LOQ
S47	*Mytilus edulis*	< LOQ	< LOQ	< LOQ	< LOQ
S48	*Mytilus edulis*	< LOQ	< LOQ	< LOQ	< LOQ
S49	*Mytilus edulis*	< LOQ	< LOQ	< LOQ	< LOQ
S50	*Mytilus edulis*	< LOQ	< LOQ	< LOQ	< LOQ
S51	*Mytilus edulis*	< LOQ	< LOQ	< LOQ	< LOQ
S53	*Mytilus edulis*	< LOQ	< LOQ	< LOQ	< LOQ
S53	*Mytilus edulis*	< LOQ	< LOQ	< LOQ	< LOQ
S54	*Mytilus edulis*	< LOQ	< LOQ	< LOQ	< LOQ

Concentrations are expressed as µg STX equiv. kg⁻¹

**Table 4:** Concentration of PSTs/TTXs in marine snails determined with the APC single-cell biosensor and LC-HRMS.

Sample	Species	Tissue	Biosensor	LC-HRMS (with TEFs)		
				∑PSTs + TTXs	∑PSTs	∑TTXs
S55	*Charonia lampas*	viscera	17,794	17,399	< LOQ	17,399
S56	*Charonia lampas*	viscera	7552	7736	< LOQ	7736
S57	*Charonia lampas*	viscera	4202	4924	< LOQ	4924
S58	*Charonia lampas*	viscera	2180	1927	< LOQ	1927
S59	*Charonia lampas*	viscera	1075	927	< LOQ	927
S60	*Charonia lampas*	flesh	< LOQ	9	< LOQ	9
S61	*Charonia lampas*	flesh	< LOQ	8	< LOQ	8
S62	*Charonia lampas*	viscera	< LOQ	5	< LOQ	5
S63	*Charonia lampas*	flesh	< LOQ	4	< LOQ	4
S64	*Charonia lampas*	viscera	< LOQ	3	< LOQ	3
S65	*Charonia lampas*	flesh	< LOQ	1	< LOQ	1
S66	*Charonia lampas*	flesh	< LOQ	< LOQ	< LOQ	< LOQ
S67	*Charonia lampas*	flesh	< LOQ	< LOQ	< LOQ	< LOQ
S68	*Charonia lampas*	flesh	< LOQ	< LOQ	< LOQ	< LOQ

Concentrations are expressed as µg STX equiv. kg⁻¹

**Table 5:** Concentration of PSTs/TTXs in clams, cockles, geoducks, and razor clams determined with the APC single-cell biosensor and LC-MS/MS.

Sample	Species	Biosensor	LC-MS/MS (with TEFs)		
			∑PSTs + TTXs	∑PSTs	∑TTXs
S69	*Spisula solida*	1365	1340	1319	21
S70	*Cerastoderma edule*	526	501	501	< LOQ
S71	*Mercenaria mercenaria*	247	222	171	51
S72	*Panopea generosa*	122	112	91	21
S73	*Cerastoderma edule*	< LOQ	8	8	< LOQ

Concentrations are expressed as µg STX equiv. kg⁻¹

**Fig. 5:**
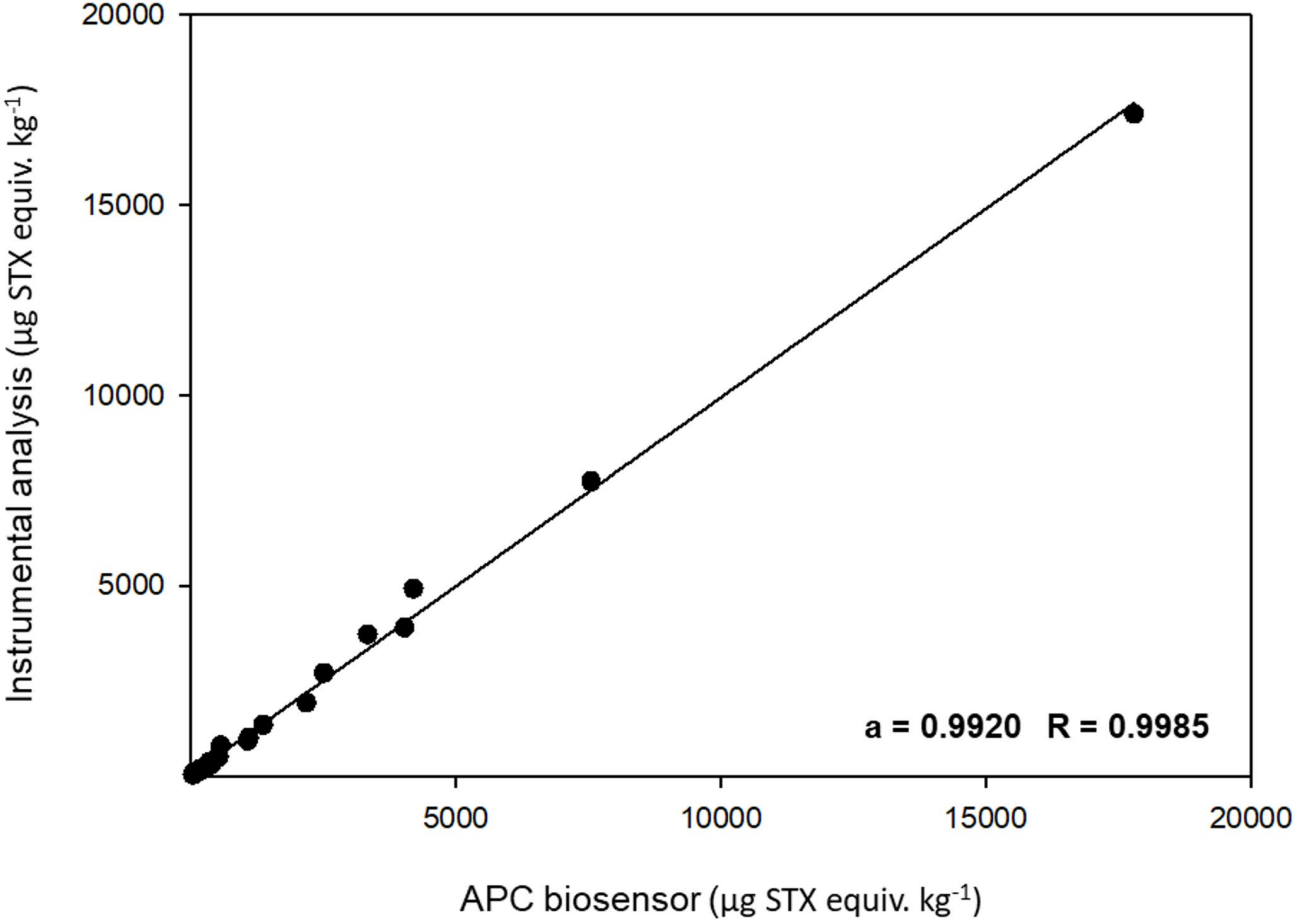
Correlation of the data obtained using the APC single-cell biosensor and instrumental analysis techniques after the application of the TEFs. The slope (a) and correlation coefficient (R) derived from the linear regression adjustment are shown

The positive biosensor results were compared with those obtained by instrumental techniques, which represent the gold standard method for the analysis of marine toxins. As illustrated in Fig. 5, the toxin concentrations determined using the biosensor correlated well (*R* = 0.9985) with those obtained by instrumental analysis techniques after application of the corresponding TEF values calculated in this work with the APC single-cell biosensing device, even though LC-MS/MS and LC-HRMS analyses were performed in different laboratories and using different protocols.

## Discussion

The single-toxin evaluations contemplated in current toxin monitoring regulations may not be adequate for human health protection since they may underestimate the overall neurotoxic risk associated with the consumption of contaminated shellfish when PSTs and TTXs co-exist. Therefore, the application of integrated analysis strategies for the simultaneous monitoring of PSTs and TTXs should be considered for a proper food safety assessment. In line with this growing need, we proposed a single-cell APC biosensing device as a promising bioanalytical tool for the simultaneous detection of PSTs and TTXs in shellfish. In Neuro-2a cells, STX and TTX contributed equally to sodium current inhibition, showing an equivalent and additive toxicity. These results align well with the observations of Finch et al. (2018), which reported that STX and TTX exert the same toxicity when administered to mice by feeding. Because the toxicities of TTX and STX are equivalent and additive, either toxin can be used as a quantitative reference for the biosensor response. In fact, the biosensor exhibited the same sensitivity towards both toxin compounds. In line with the scientific opinions recommending the inclusion of TTXs within the group of PSTs (Finch et al. 2018), STX was selected as the reference compound instead of TTX. For this work, the regulatory limit of 800 µg STX equiv. kg⁻¹ (Regulation (EC) No 853/2004) was adopted as the threshold for determining whether samples were safe for human consumption. It should be noted, however, that given the excellent LOD and LOQ achieved with the biosensor, the system can be readily adapted in the future in response to potential changes or revisions in regulatory limits and/or safety standards.

In addition to STX and TTX, the ability of the single-cell APC biosensing device to detect toxin analogues was also evaluated. In general terms, the toxicities observed in this work for PSTs were consistent with those proposed by EFSA (EFSA, 2009), although some discrepancies were noted. For example, EFSA classifies dcSTX as being as toxic as STX, whereas it exhibited a TEF_APC_ of 0.029 in this work. This discrepancy has been previously reported (Alkassar et al. 2024a; Alonso et al. 2016) and is likely attributable to differences in the expression of VGSC isoforms across the different toxicological models used for the determination of the TEFs (Alonso et al. 2016). Another discrepancy concerned NEO, which EFSA also classifies as equipotent to STX but showed a higher toxicity in this work (TEF_APC_ = 1.546). By contrast, GTX2&3, dcGTX2&3, GTX5 and GTX6 exhibit slightly lower TEF_APC_ values in this work than those proposed by EFSA It is important to note that TEFs may vary depending on the toxin standards employed, the analytical or bioassay techniques used, minor protocol variations, and intrinsic interlaboratory variability. Consequently, TEFs should not be regarded as universal constants, underscoring the need for continued evaluation of the toxicological properties of toxin analogues. In line with this, a FAO/WHO report proposed revised TEFs for several STX analogues, identifying dcSTX as less toxic and NEO as more toxic than originally reported (FAO/WHO, 2016), which is aligned with the observations from this work. Focusing on the molecular structure of the STX analogues, it can be inferred that the substituent at position R_4_ plays a critical role in the toxicity of PSTs on Neuro-2a cells. The analogues bearing a carbamoyl group at R_4_ position (i.e., STX, NEO, GTX1&4, and GTX2&3) exhibit higher toxicity than those containing an *N*-sulfocarbamoyl group (i.e., GTX5, GTX6, C1&2, and C3&4) or a hydroxyl group (i.e., dcSTX and dcGTX2&3). In contrast, the substituents at positions R_1_-R_3_ appear to exert a more modest influence on overall toxicity, while remaining functionally relevant. For example, the higher toxicity of NEO compared with STX suggests that the introduction of a hydroxyl group at position R_1_, may enhance the toxin potency by modulating the orientation or stabilisation of the molecule within the VGSC binding site. Regarding TTX analogues, they were previously determined in Neuro-2a cells using the same APC single-cell biosensor applied in this study (Reverté et al. 2024). Because TTX and STX have equivalent toxicity, using STX instead of TTX as the reference compound does not alter the previously established TEF_APC_ values for TTX analogues. Therefore, the TEF_APC_ values remain as reported by Reverté et al. (2024). These results demonstrate that, in addition to STX and TTX, the biosensor can also detect their toxic analogues and, therefore, provide a composite response product of the individual toxicities of all toxin analogues present in a sample. However, this would be only valid if shellfish matrix components are not affecting the detection.

Shellfish matrix components may impact APC sodium current recordings not only by inducing a non-specific blockage of the VGSCs (i.e., physical pore obstruction) but also by affecting other cellular structures or metabolic processes that could indirectly influence sodium fluxes. In toxin-free samples, where matrix is present but PSTs and TTXs are absent, such interference could lead to false-positive results. In general terms, the matrix effects were comparable among the different types of shellfish tested. However, it should be highlighted that gastropod samples required an SPE clean-up step to mitigate matrix interference. This was necessary because marine snail matrices are known to exert stronger matrix effects than mussels or oysters, as previously reported (Han et al. 2023). Based on these results and considering that matrix effects may vary among shellfish species and/or specimens (Campàs et al. 2020), we established that a conservative shellfish tissue concentration of 10 mg mL⁻¹ should not induce any non-specific blockage of the sodium currents in Neuro-2a cells. Accordingly, all shellfish samples should be diluted at least 200-fold before analysis to avoid false-positive results. In addition to the non-specific blockage, shellfish matrix components may also hinder PSTs or TTXs from binding their toxin binding site in VGSCs, leading to false-negative results. However, the good recoveries observed indicated that by analysing the samples at a tissue equiv. concentration of 10 mg mL⁻¹ or lower, the biosensor avoids not only false-positive but also false-negative results.

To validate the single-cell APC biosensing device, a total of 73 shellfish samples collected from several European regions and Mexico were analysed as part of an intralaboratory validation trial. The sample set included oysters, mussels, marine snails, clams, cockles, geoducks, and razor clams. Overall, the toxin contents among the different shellfish groups were within a comparable range, but substantially lower than those detected in marine snails. All specimens in the marine snail group belonged to *Charonia lampas*, a species known for being responsible for the first documented case of TTX poisoning linked to shellfish in Europe (Rodríguez et al. 2008; Fernández-Ortega et al. 2010). In this species, the highest toxin levels, comparable to those reported in pufferfish (Alkassar et al. 2023; Reverté et al. 2024), are present predominantly in the visceral tissues. Some authors have suggested that, as practiced for pufferfish in Japan (Katikou et al. 2022), the removal of the visceral parts could reduce the risk of poisoning (Pais et al. 2025). However, such processing is technically challenging and may result in cross-contamination of other tissues. Regarding the other shellfish species, although toxin levels were substantially lower than those observed in marine snails, they were not negligible, highlighting the need for continued surveillance and risk assessment across all shellfish groups. Importantly, a good agreement between APC and reference analytical techniques after the application of the corresponding TEFs was obtained, indicating that the biosensor provides a reliable and accurate estimation of the overall toxicological risk of the sample.

Overall, based on the results obtained with the APC biosensor, ten samples (S01, S41, S42, S43, S55, S56, S57, S58, S59, and S69) exceeded the established safety threshold of 800 µg STX equiv. kg⁻¹ and would therefore be deemed unsafe for human consumption (Regulation (EC) No 853/2004). However, if these samples were assessed exclusively for PSTs, only five (S01, S41, S42, S43, and S69) would be considered life-threatening, while the remaining five (S55, S56, S57, S58, and S59) would be classified as safe. In this particular case, as indicated by the instrumental analysis results, this discrepancy was due the presence of TTXs but no PSTs in the samples. However, since the coexistence of PSTs and TTXs was observed in other samples (S01, S42, S43, S69, S71 and S72), a scenario of additive toxicity should not be dismissed. This underscores the importance of including TTXs in official PST monitoring to ensure accurate risk assessment and effective public health protection with tools as that proposed in this work.

## Conclusions

In this work, we present a cutting-edge APC single-cell biosensing device for the simultaneous detection of PSTs and TTXs in shellfish. The biosensor was capable of detecting not only parent STX and TTX, but also a broad range of their toxic analogues, providing an integrated assessment of the overall toxicity of the sample. Moreover, the possibility of using a unified protocol to co-extract PSTs and TTXs together with the minimal matrix-related interferences affecting toxin detection, substantially contribute to simplify the whole analytical procedure.

This high-throughput biosensing platform is well suited for routine toxin monitoring in decentralised laboratories, as the entire analytical workflow is fully automated, rendering the system user-friendly and suitable for non-specialist operation. Although the acquisition and maintenance costs of an APC device are comparable to those of advanced instrumental techniques such as LC-MS/MS, LC-HRMS, or HPLC-FLD, the proposed approach can be more cost-effective in routine analysis. Data interpretation is more straightforward, and the method does not require highly specialised expertise. Compared with other toxicological alternatives, such as the conventional cell-based assay, the APC biosensor offers faster turnaround times, higher standardisation, and full automation, further supporting its implementation in laboratories with limited specialist staff.

Following validation with more than 70 contaminated shellfish samples with diverse PST/TTX profiles, the biosensor demonstrated strong potential as an integrative approach for toxin monitoring in support of food safety and public health protection. Importantly, the current EU reference method for PST determination does not enable the detection of TTXs. Therefore, the implementation of complementary or alternative methodologies capable of simultaneously detecting both toxin groups, such as the one proposed in this work, would substantially strengthen official control programmes and enhance consumer protection.

## Data Availability

Data will be made available on request.
